# Implication of diabetic status on platelet reactivity and clinical outcomes after drug-eluting stent implantation: results from the PTRG-DES consortium

**DOI:** 10.1186/s12933-023-01976-4

**Published:** 2023-09-07

**Authors:** Ki-Hyun Jeon, Young-Hoon Jeong, In-Ho Chae, Byeong-Keuk Kim, Hyung Joon Joo, Kiyuk Chang, Yongwhi Park, Young Bin Song, Sung Gyun Ahn, Sang Yeub Lee, Jung Rae Cho, Ae-Young Her, Hyo-Soo Kim, Moo Hyun Kim, Do-Sun Lim, Eun-Seok Shin, Jung-Won Suh, On Behalf of the PTRG-DES Consortium Investigators

**Affiliations:** 1 Department of Internal Medicine, Department of Cardiology, Seoul National University College of Medicine, Seoul National University Bundang Hospital, Seongnam, South Korea; 2 CAU Thrombosis and Biomarker Center, Department of Internal Medicine, Chung-Ang University Gwangmyeong Hospital, Chung-Ang University College of Medicine, Gwangmyeong, Seoul, South Korea; 3 Severance Cardiovascular Hospital, Yonsei University College of Medicine, Seoul, South Korea; 4 Department of Cardiology, Cardiovascular Center, Korea University Anam Hospital, Korea University College of Medicine, Seoul, South Korea; 5 Division of Cardiology, Department of Internal Medicine, College of Medicine, The Catholic University of Korea, Seoul, South Korea; 6 Department of Internal Medicine, Cardiovascular Center, Gyeongsang National University School of Medicine, Gyeongsang National University Changwon Hospital, Changwon, South Korea; 7 Division of Cardiology, Department of Medicine, Samsung Medical Center, Sungkyunkwan University School of Medicine, Seoul, South Korea; 8 Department of Cardiology, Yonsei University Wonju Severance Christian Hospital, Wonju, South Korea; 9 Division of Cardiology, Department of Internal Medicine, Chung-Ang University Gwangmyeong Hospital, Chung-Ang University College of Medicine, Gwangmyeong, South Korea; 10 Cardiology Division, Department of Internal Medicine, Kangnam Sacred Heart Hospital, Hallym University College of Medicine, Seoul, South Korea; 11 Division of Cardiology, Department of Internal Medicine, Kangwon National University School of Medicine, Chuncheon, South Korea; 12 Department of Internal Medicine and Cardiovascular Center, Seoul National University Hospital, Seoul, South Korea; 13 Department of Cardiology, Dong-A University Hospital, Busan, South Korea; 14 Division of Cardiology, Ulsan University Hospital, University of Ulsan College of Medicine, Ulsan, South Korea

**Keywords:** Platelet reactivity, Diabetes mellitus, Stenting, Hemoglobin _A1c_, Insulin

## Abstract

**Background:**

Diabetes mellitus (DM) is associated with thrombogenicity, clinically manifested with atherothrombotic events after percutaneous cutaneous intervention (PCI). This study aimed to investigate association between DM status and platelet reactivity, and their prognostic implication in PCI-treated patients.

**Methods:**

The Platelet function and genoType-Related long-term Prognosis-Platelet Function Test (PTRG-PFT) cohort was established to determine the linkage of platelet function test (PFT) with long-term prognosis during dual antiplatelet therapy including clopidogrel in patients treated with drug-eluting stent (DES). We assessed platelet reactivity using VerifyNow and ‘high platelet reactivity (HPR)’ was defined as ≥ 252 P2Y12 reaction unit (PRU). Major adverse cardiac and cerebrovascular event (MACCE) was a composite of all-cause death, myocardial infarction, stent thrombosis or stroke.

**Results:**

Between July 2003 and Aug 2018, DES-treated patients with available PFT were enrolled (n = 11,714). Diabetic patients demonstrated significant higher levels of platelet reactivity (DM vs. non-DM: 225.7 ± 77.5 vs. 213.6 ± 79.1 PRU, *P* < 0.001) and greater prevalence of HPR compared to non-diabetic patients (38.1% vs. 32.0%, *P* < 0.001). PRU level and prevalence of HPR were significantly associated with insulin requirement and Hb_A1c_ level, as well as diabetic status. DM status and HPR phenotype had a similar prognostic implication, which showed the synergistic clinical impact on MACCE. Association between PRU level and MACCE occurrence seemed higher in diabetic vs. non-diabetic patients. In non-DM patients, HPR phenotype did not significantly increase the risk of MACCE (adjusted hazard ratio [HR_adj_]: 1.073; 95% confidence interval [CI]: 0.869–1.325; *P* = 0.511), whereas HPR was an independent determinant for MACCE occurrence among diabetic patients (HR_adj_: 1.507; 95% CI: 1.193–1.902; *P* < 0.001).

**Conclusion:**

The levels of on-clopidogrel platelet reactivity are determined by diabetic status and the severity of DM. In addition, HPR phenotype significantly increases the risk of MACCE only in diabetic patients.

**Clinical trial registration:**

URL: https://www.clinicaltrials.gov. Unique identifier: NCT04734028.

**Supplementary Information:**

The online version contains supplementary material available at 10.1186/s12933-023-01976-4.

## Introduction

Diabetes mellitus (DM) increases the risk of morbidity and mortality due to atherosclerotic cardiovascular disease (ASCVD). Many studies have shown that DM patients without prior ASCVDs are at the same risk for cardiovascular events as patients without diabetes with a history of earlier cardiovascular events [1, 2]. In patients with diabetes, hyperglycemia, insulin resistance, glucose variability, and systemic inflammation directly or indirectly contribute to the pathogenesis of atherosclerosis and lead to micro- and macro-vascular complications [3–6]. In addition to atherogenicity, DM is clinically manifested by high rate of acute thromboembolic events, including acute myocardial infarction (AMI), stroke and venous thromboembolism [7, 8]. These findings can be related with increased thrombogenicity owing to platelet hyperreactivity, activation of coagulation factors and hypo-fibrinolysis [9, 10].

High platelet reactivity (HPR) phenotype measured by platelet function test (PFT) is a well-established predictor of major cardiovascular events (MACEs) after percutaneous coronary intervention (PCI) [11–13]. Platelet reactivity in diabetic patients can increase according to metabolic abnormalities including hyperglycemia [14], insulin resistance/deficiency [15], oxidative stress, and endothelial dysfunction [16, 17]. Moreover, it can impair the responsiveness to antiplatelet therapy [18, 19]. The present study aimed to investigate the association between diabetic conditions and the level of platelet reactivity, and their clinical implication in a large-scale cohort including patients with significant coronary artery disease (CAD).

## Methods

### Study design and patients

The PTRG-DES (Platelet function and genoType-Related long-term proGnosis in Drug Eluting Stent-treated patients) consortium is an investigator-initiative nationwide multicenter observational registry endorsed by the Korean Society of Interventional Cardiology, specifically designed to determine the relationship between platelet reactivity/genotype and subsequent clinical events in East Asian patients after uneventful drug-eluting stent (DES) implantation [20].

In total, nine prospective registries from 32 Korean academic centers have joined the PTRG-DES consortium, contributing data from 13,160 DES patients treated between July 2003 and August 2018. Consecutive patients who were treated with DES and had been adequately administered both aspirin and clopidogrel were eligible for enrollment, irrespective of patients’ medical conditions or complexity of coronary artery lesions. The major exclusion criteria were the occurrence of a major complication during the procedure, fibrinolytic therapy, and the need for oral anticoagulants. DM was classified by one of the followings: (1) a history of diabetes, regardless of duration of disease, or need for antidiabetic agents; (2) a fasting blood glucose ≥ 126 mg/dl; or (3) glycosylated hemoglobin (Hb_A1c_) ≥ 6.5% [21].

The institutional review board of each participating center approved the registry and waived the requirement for written informed consent for access to an institutional registry. The study was performed in accordance with the Good Clinical Practice Guidelines and the principles of the Declaration of Helsinki.

### Platelet function test

We obtained 11,714 PFT results (PTRG-PFT cohort), in which platelet reactivity was measured after an adequate period to ensure a full antiplatelet effect, using the VerifyNow assay (Accriva, San Diego, CA, USA) [22]. The measurement protocol followed the manufacturer’s recommendations, and the details are described elsewhere [23]. Aspirin was given as either: (1) a coated oral dose of 300 mg for at least 6 h; or (2) a dose of 100 mg at least 5 days before PCI. Clopidogrel was given as either (1) a dose of 600 mg at least 6 h; (2) a dose of 300 mg at least 12 h; or (3) a dose of 75 mg for at least 5 days before PCI. If eptifibatide or tirofiban was used during PCI, a 24-hr washout period was required before VerifyNow testing. No patients receiving abciximab were enrolled because of a long washout period.

The levels of platelet reactivity on clopidogrel and aspirin were reported as ‘P2Y12 reaction unit (PRU)’ and ‘aspirin reaction unit (ARU)’, respectively. We assessed PRUs as continuous and categorical measures. Additionally, the cutoffs of ‘HPR to ADP’ and ‘HPR to arachidonic acid (AA)’ were defined as ‘≥ 252 PRU’ and ‘≥ 414 ARU’ according to our previous report [20].

### Clinical outcomes

The primary endpoint was the occurrence of major adverse cardiac and cerebrovascular events (MACCE) including all-cause death, myocardial infarction (MI), definite stent thrombosis (ST) or stroke for 5 years post-PCI. In addition, major bleeding was defined as Bleeding Academic Research Consortium (BARC) bleeding type 3–5 [24].

All deaths were considered to be due to cardiovascular (CV) cause unless a definite non-CV cause could be established. AMI was defined as increased cardiac troponin values with ischemic symptoms or ischemic changes on electrocardiogram or imaging evidence of recent loss of viable myocardium or new regional wall motion abnormalities that were not related to the procedure [25]. Stroke included any new embolic, thrombotic, or hemorrhagic stroke events with neurologic deficits that persisted for at least 24 h. An independent clinical event committee masked to the VerifyNow results adjudicated all clinical events using the original source documents.

### Statistical analysis

The Kolmogorov–Smirnov test was performed to analyze the normal distribution of continuous variables. Continuous variables were expressed as mean ± standard deviation (SD) or as median [interquartile range (IQR)], while categorical variables were presented as absolute numbers and frequencies (%). Student’s unpaired t-test and the Mann-Whitney U test were used for evaluating the parametric and the non-parametric continuous variables, respectively. Categorical variables were compared using the Pearson chi-square test or Fisher’s exact test when the Cochran rule was not met. Univariate and multivariate Cox proportional hazard analyses were performed to identify proportional hazard risk for clinical events according to DM status and/or HPR phenotype. To adjust for potential confounders (age, sex, body mass index, index MI presentation, hypertension, dyslipidemia, smoking, DM, chronic kidney disease [CKD], anemia, congestive heart failure, previous PCI, previous stroke, multivessel disease, PCI for left main or left anterior descending artery, and DES generation), variables with *P* < 0.1 in univariate analysis were then entered into multivariate logistic augmented backwards regression analysis providing odds ratio (OR) and 95% confidence intervals (CIs). Statistical significance was set at *P-* value < 0.05. All statistical analyses were performed using IBM/SPSSv24.0 (IBM/SPSS, Chicago, IL, USA) and RStudio (Integrated Development Environment for R. RStudio, PBC, Boston, MA, USA).

## Results

### Baseline characteristics of the study population

From the PTRG-PFT cohort (n = 11,714), the level of platelet reactivity was 217.8 ± 78.7 PRU and prevalence of HPR (≥ 252 PRU) was 34.2% (n = 4,001). Approximately 35% of patients (n = 4,057) had diabetes (Supplement Fig. 1). Table 1 showed the baseline characteristics according to the presence of DM. Compared to non-diabetic patients, diabetic patients were older (DM vs. non-DM: 65.5 ± 10.0 vs. 63.8 ± 11.3, *P* < 0.001), the proportion of female was higher (34.8% vs. 30.7%, *P* < 0.001), and the prevalence of hypertension (70.9% vs. 54.5%, *P* < 0.001) and hyperlipidemia (65.9% vs. 63.7%, *P* = 0.019) was higher, while the proportion of current smokers was lower (25.7% vs. 29.3%, *P* < 0.001).

**Fig. 1:**
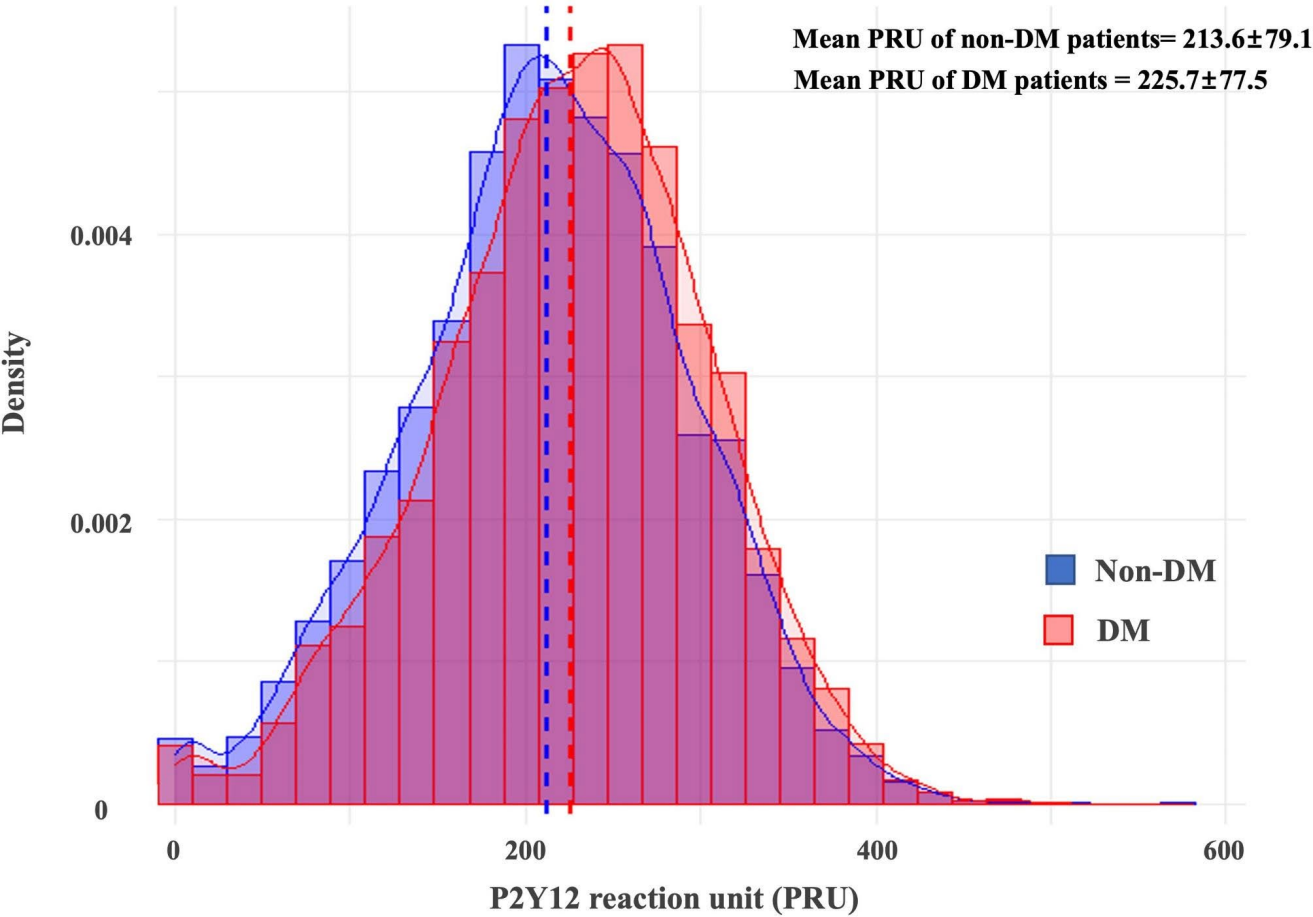
Distribution of PRU according to DM status DM: diabetes mellitus; PRU: P2Y12 reaction unit

**Table 1:** Baseline characteristics of study population according to DM

	Non-DM (N = 7,657)	DM (N = 4,057)	*P* value
Index presentation, n (%)			< 0.001
Stable angina	3,071 (40.1)	1,839 (45.3)	
Unstable angina	2,257 (29.5)	1,209 (29.8)	
Non-ST-segment elevation MI	1,252 (16.4)	608 (15.0)	
ST-segment elevation MI	1,077 (14.1)	401 (9.9)	
Age, years	63.8 ± 11.3	65.5 ± 10.0	< 0.001
Male, n (%)	5,036 (69.3)	2,645 (65.2)	< 0.001
Body mass index, kg/m^2^	24.4 ± 3.09	24.8 ± 3.14	< 0.001
Risk factors, n (%)^*^			
Hypertension	4172 (54.5%)	2877 (70.9%)	< 0.001
Dyslipidemia	4880 (63.7%)	2675 (65.9%)	0.019
Smoking	2241 (29.3%)	1044 (25.7%)	< 0.001
Chronic kidney disease	1292 (16.9%)	1140 (28.1%)	< 0.001
Current dialysis	61 (0.8%)	101 (2.5%)	< 0.001
Anemia	1618 (21.1%)	1303 (32.1%)	< 0.001
Previous history, n (%)			
History of peripheral artery disease	886 (11.6%)	567 (14.0%)	< 0.001
History of congestive heart failure	555 (7.2%)	325 (8.0%)	0.146
Previous MI	504 (6.6%)	335 (8.3%)	0.001
Previous PCI	906 (11.8%)	662 (16.3%)	< 0.001
Previous CABG	71 (0.9%)	79 (1.9%)	< 0.001
Previous stroke	467 (6.1%)	346 (8.5%)	< 0.001
Laboratory measurements			
VerifyNow PRU	213.6 ± 79.1	225.7 ± 77.5	< 0.001
LV ejection fraction, %	59.1 ± 10.2	58.1 ± 11.3	< 0.001
WBC, x10^3^/mm^3^	7.8 ± 3.0	7.9 ± 2.8	0.378
Hemoglobin, g/dL	13.8 ± 1.8	13.2 ± 2.0	< 0.001
Platelet, x10^3^/mm^3^	233.9 ± 70.6	233.2 ± 75.7	0.657
GFR, mL/min/1.73 m^2^ (MDRD)	80.9 ± 25.2	74.5 ± 29.8	< 0.001
HbA_1c_, %	6.0 ± 0.9	7.5 ± 1.6	< 0.001
Total cholesterol, mg/dL	178.9 ± 43.9	165.9 ± 43.4	< 0.001
LDL-cholesterol, mg/dL	110.1 ± 38.1	98.9 ± 36.2	< 0.001
HDL-cholesterol, mg/dL	44.6 ± 12.0	42.4 ± 11.2	< 0.001
Triglyceride, mg/dL	140.8 ± 99.7	147.7 ± 95.4	< 0.001
Angiographic feature			
ACC/AHA lesion, n (%)			0.001
A/B1 type	3509 (45.8%)	1729 (42.6%)	
B2/C type	4148 (54.2%)	2328 (57.4%)	
Number of diseased vessels, n (%)			<0.001
One	4840 (63.2%)	2330 (57.4%)	
Two	1926 (25.2%)	1113 (27.4%)	
Three	891 (11.6%)	614 (15.1%)	
Multivessel disease, n (%)	2817 (36.8%)	1727 (42.6%)	< 0.001
Bifurcation lesion, n (%)	844 (11.0%)	519 (12.8%)	0.005
Chronic total occlusion lesion, n (%)	496 (6.5%)	325 (8.0%)	0.002
Procedural data			
Multivessel PCI, n (%)	1780 (23.2%)	1137 (28.0%)	< 0.001
Treated lesions, n (%)			
Left main coronary artery	391 (5.1%)	181 (4.5%)	0.135
Left anterior descending artery	4532 (59.2%)	2428 (59.8%)	0.502
Left circumflex artery	2170 (28.3%)	1264 (31.2%)	0.002
Right coronary artery	2829 (36.9%)	1631 (40.2%)	0.001
PCI for left main or left anterior descending artery, n (%)	4790 (62.6%)	2537 (62.5%)	0.996
Stent type, n (%) ^**†**^			0.912
1st generation DES	615 (8.0%)	329 (8.1%)	
2nd generation DES	7042 (92.0%)	3728 (91.9%)	
Number of stents, n	1.6 ± 0.8	1.6 ± 0.8	< 0.001
Stent length, mm	34.9 ± 22.1	37.6 ± 23.2	< 0.001
Stent diameter, mm	3.0 ± 0.4	3.0 ± 0.4	< 0.001
Concomitant medications, n (%)			
Aspirin	7478 (97.7%)	3931 (96.9%)	0.015
Clopidogrel	7657 (100.0%)	4057 (100.0%)	1.000
Cilostazol	762 (10.0%)	457 (11.3%)	0.029
Beta blocker	4436 (57.9%)	2233 (55.0%)	0.003
Angiotensin blockade	4393 (57.4%)	2534 (62.5%)	< 0.001
Calcium channel blocker	1761 (23.0%)	1056 (26.0%)	< 0.001
Statin	6832 (89.2%)	3547 (87.4%)	0.004
Proton pump inhibitor	1347 (17.6%)	644 (15.9%)	0.020

Continuous variables were expressed in mean ± SD or median (IQR) as indicated

ACC: American College of Cardiology; AHA: American Heart Association; CABG: coronary artery bypass graft; DES: drug eluting stent; GFR: glomerular filtration rate; HbA_1c_, hemoglobin A_1c_; HDL: high density lipoprotein; LDL: low density lipoprotein; LV: left ventricular; MDRD: Modification of Diet in Renal Disease; MI: myocardial infarction; PCI: percutaneous coronary intervention; PRU: P2Y12 Reaction Unit; WBC: white blood cell

^*^Hypertension was diagnosed by one of the followings: (1) history of hypertension diagnosed and treated with medication, diet and/or exercise; (2) blood pressure greater than 140 mmHg systolic or 90 mmHg diastolic on at least 2 occasions; or (3) currently on antihypertensive pharmacologic therapy.; Dyslipidemia was diagnosed by one of followings: (1) total cholesterol ≥ 200 mg/dl; (2) LDL cholesterol ≥ 130 mg/dl; (3) HDL cholesterol < 40 mg/dl; or (4) triglycerides ≥ 150 mg/dl; Current smoker was defined as use of tobacco within one year of this admission; Diabetes mellitus was diagnosed by one of the followings: (1) a history of diabetes, regardless of duration of disease, or need for antidiabetic agents; (2) a fasting blood glucose ≥ 126 mg/dl; or (3) glycosylated hemoglobin ≥ 6.5; Chronic kidney disease was diagnosed by one of the followings: (1) GFR < 60 mL/min/1.73m^2^ (MDRD); (2) on dialysis; or (3) history of a renal transplantation; Anemia was defined as hemoglobin level < 12 g/dl in women and 13 g/dl in men

^**†**^First-generation DES indicated durable polymer-based paclitaxel-eluting stents (PES: Taxus, Pico) or sirolimus-eluting stent (SES: Cypher); Second-generation DES indicated next-generation DESs including everolimus-eluting stent (EES: Promus, Xience), zotarolimus-eluting stent (ZES: Endeavor, Resolute, Onyx), biolimus-eluting stent (BES: Biolimus A9), and plymer-free SES.; If a patient were treated with first- and second-generation DESs together, this patient was considered as implantation with first generation DES.

### Platelet reactivity and prevalence of HPR according to diabetic condition

The levels of platelet reactivity in diabetic patients were significantly higher than that of non-diabetic patients (DM vs. non-DM: 225.7 ± 77.5 vs. 213.6 ± 79.1 PRU, *P* < 0.001 and 448.2 ± 72.3 vs. 442.1 ± 67.7 ARU, *P* < 0.001) (Fig. 1; Table 2). In addition, prevalence of HPR to ADP phenotype was higher in DM patients compared with non-DM subjects (HPR to ADP: 38.1% vs. 32.0%, *P* < 0.001 and HPR to AA: 53.7% vs. 51.6%, *P* = 0.090). Even for the diabetic patients, the levels of platelet reactivity and prevalence of HPR phenotypes varied depending on the need for insulin (Table 2); DM patients on insulin (N = 270, 6.7% of DM patients) showed the highest levels of platelet reactivity compared with other groups (DM on insulin vs. DM without insulin vs. non-DM: 44.0% vs. 37.7% vs. 32.0%, *P* < 0.001) Furthermore, we divided enrolled patients into the three groups according to on-admission Hb_A1c_ level (available data: n = 4,095); Hb_A1c_ < 6.5% (n = 2,541, 62.1%), 6.5–8.5% (n = 1,192, 29.1%) and > 8.5% (n = 362, 8.8%). Hb_A1c_ level showed the weak positive relationship with levels of platelet reactivity (vs. PRU: *r* = 0.065, *P* < 0.001 and vs. ARU: *r* = 0.049, *P* = 0.101, respectively) (Supplement Fig. 2). Therefore, PRU level proportionally increased across the Hb_A1c_ group (216.1 ± 82.2 vs. 226.8 ± 81.6 vs. 229.4 ± 81.4 PRU, *P* < 0.001). The risk of HPR increased with a significant correlation with Hb_A1c_ between Hb_A1c_ 6.5% and 8.5%, while there was no significant increase in the HPR risk below 6.5% and above 8.5%.

**Table 2:** Platelet reactivity and prevalence of HPR according to DM and DM severity (total n = 11,714)

	Non-DM (N = 7,657)		DM (N = 4,057)		*P*-value
**PRU** (n = 11,714)	213.6 ± 79.1		225.7 ± 77.5		< 0.001
**HPR to ADP**	32.0%		38.1%		< 0.001
**ARU** (n = 7,162)	442.1 ± 67.7		448.2 ± 72.3		< 0.001
**HPR to arachidonic acid**	51.6%		53.7%		0.090
	**Non-DM** **(N = 7,657)**	**DM without insulin** **(N = 3,787)**		**DM on insulin** **(N = 270)**	***P*** **-value**
**PRU** (n = 11,714)	213.6 ± 79.1	225.3 ± 77.8		230.7 ± 73.2	< 0.001
**HPR to ADP**	32.0%	37.7%		44.0%	0.001
**ARU** (n = 7,162)	442.1 ± 67.7	446.7 ± 72.2		462.7 ± 74.0	< 0.001
**HPR to arachidonic acid**	51.6%	52.8%		63.8%	0.002
	**HbA1c < 6.5** **(N = 2,541)**	**6.5 ≤ HbA1c ≤ 8.5** **(N = 1,192)**		**HbA1c > 8.5** **(N = 362)**	***P*** **-value**
**PRU** (n = 4,095)	216.1 ± 82.2	226.8 ± 81.6		229.4 ± 81.4	< 0.001
**HPR to ADP**	34.6%	40.2%		40.3%	0.001
**ARU** (n = 1,115)	436.4 ± 67.1	439.5 ± 70.6		449.7 ± 72.4	0.187
**HPR to arachidonic acid**	45.9%	47.1%		54.0%	0.314

Continuous variables were expressed in mean ± SD.

‘HPR to ADP’ indicates ‘≥ 252 PRU’ and ‘HPR to arachidonic acid (AA)’ indicates ‘≥ 414 ARU’.

AA: arachidonic acid; ADP: adenosine diphosphate; ARU: aspirin reaction unit; DM: diabetes mellitus; HbA1c: hemoglobin A1c; HPR: high platelet reactivity; PRU: P2Y12 reaction unit

**Fig. 2:**
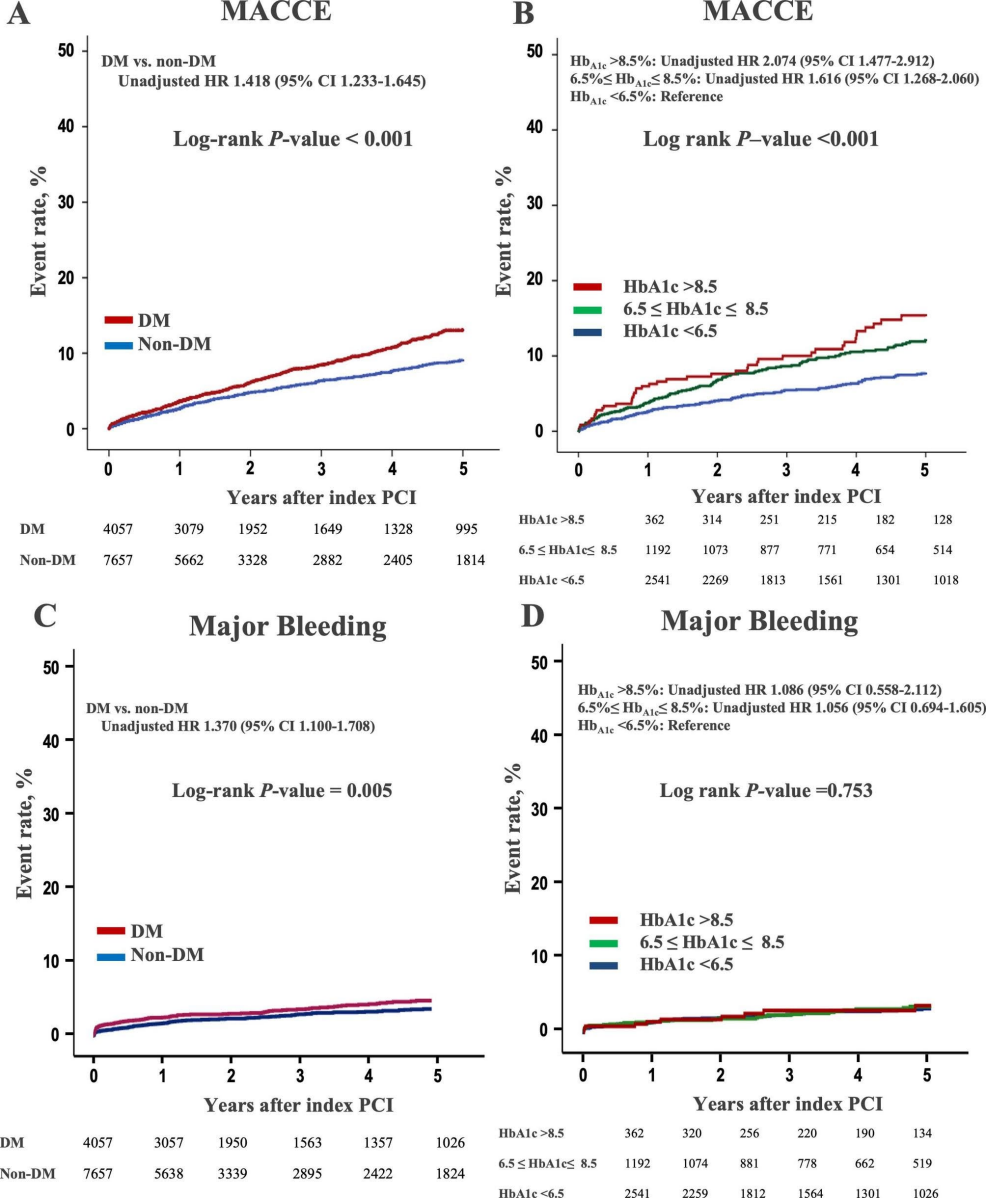
Kaplan–Meier curves of MACCE and major bleeding rate according to diabetic condition. **A** 5-year MACCE with and without DM; **B** 5-year MACCE according to index Hb A1c; **C** 5-year major bleeding with and without DM; **D** 5-year major bleeding according to index Hb A1c Hb A1c: hemoglobin A1c; MACCE: major cardiac and cerebrovascular event

### Prognostic impact of platelet reactivity according to diabetic condition

During the median follow-up of 37.6 months (IQR, 12.0–60.8), a total of 709 MACCEs (6.1%) (392 deaths [3.3%], 172 non-fatal MI [1.5%], 62 ST [0.5%] and 181 non-fatal stroke [1.5%]), and 324 cases of major bleeding (2.8%) occurred. During 5-year follow-up, the MACCE rate in diabetic patients was higher than that in non-diabetic patients (7.7% vs. 5.2%, unadjusted hazard ratio [HR] 1.418, 95% CI: 1.233–1.645, *P* < 0.001), and the index Hb_A1c_ levels were also related with the risk of MACCE (Fig. 2A and B). The rate of major bleeding was also higher in DM patients (3.4% vs. 2.4%, unadjusted HR 1.370, 95% CI: 1.100-1.708, *P* = 0.005) (Fig. 2C and D).

**Fig. 3:**
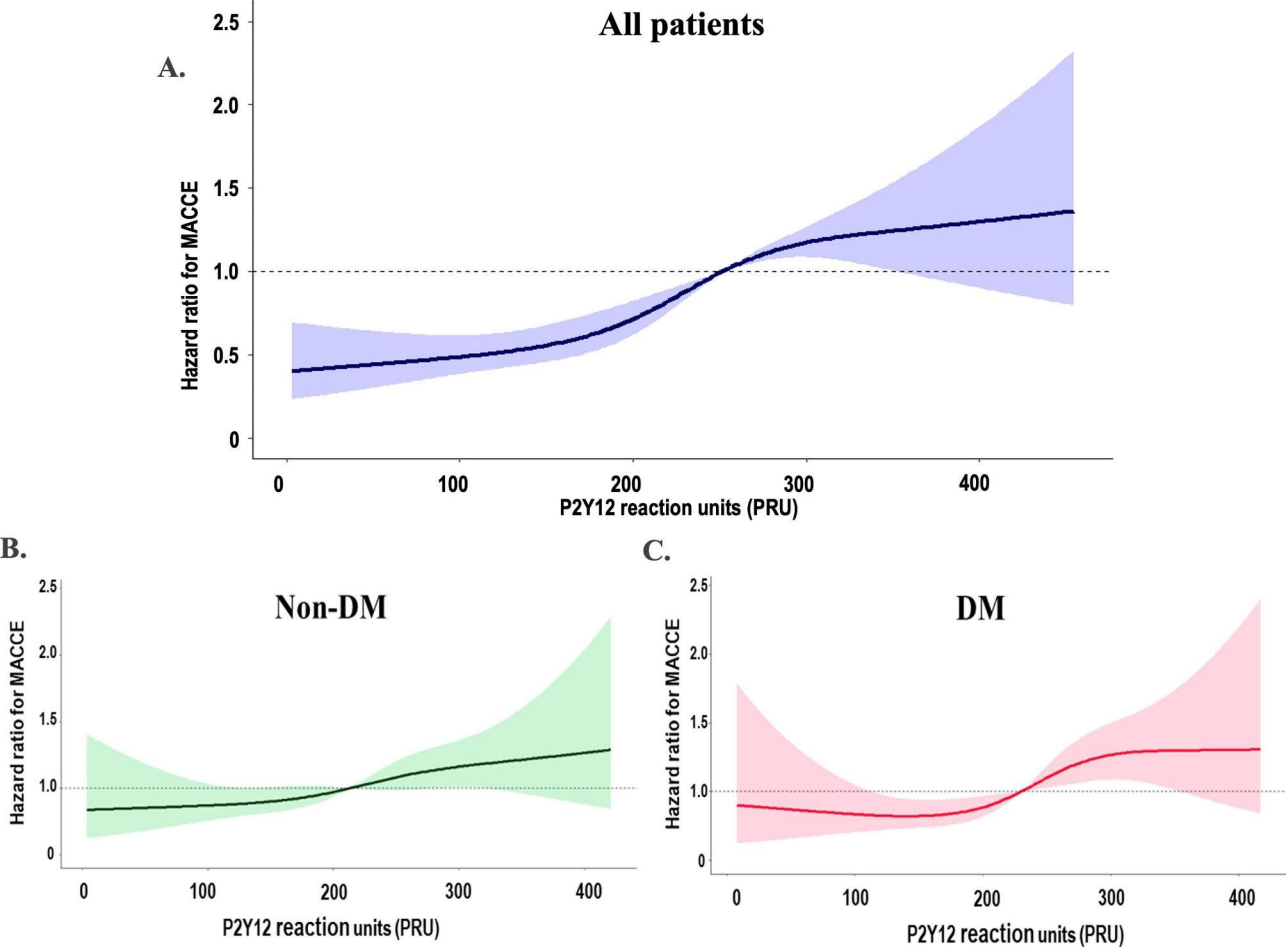
Relative hazard ratio for MACCE according to PRU level by restricted cubic spline curve. **A** all patients; **B** non-diabetic patients; **C** diabetic patients MACCE: major cardiac and cerebrovascular event; PRU: P2Y12 reaction unit

Figure 3 showed relative HR for MACCE occurrence according to PRU level. Among the total cohort, the cutoff of platelet reactivity for increasing the risk of MACCE was observed around HPR (PRU ≥ 252). Association between PRU level and MACCE occurrence appeared closer in DM patients compared with non-DM subjects. The cutoff of PRU for MACCE occurrence seemed to be similar between the groups.

### Prognostic impact of HPR phenotype according to diabetic condition

**Fig. 4:**
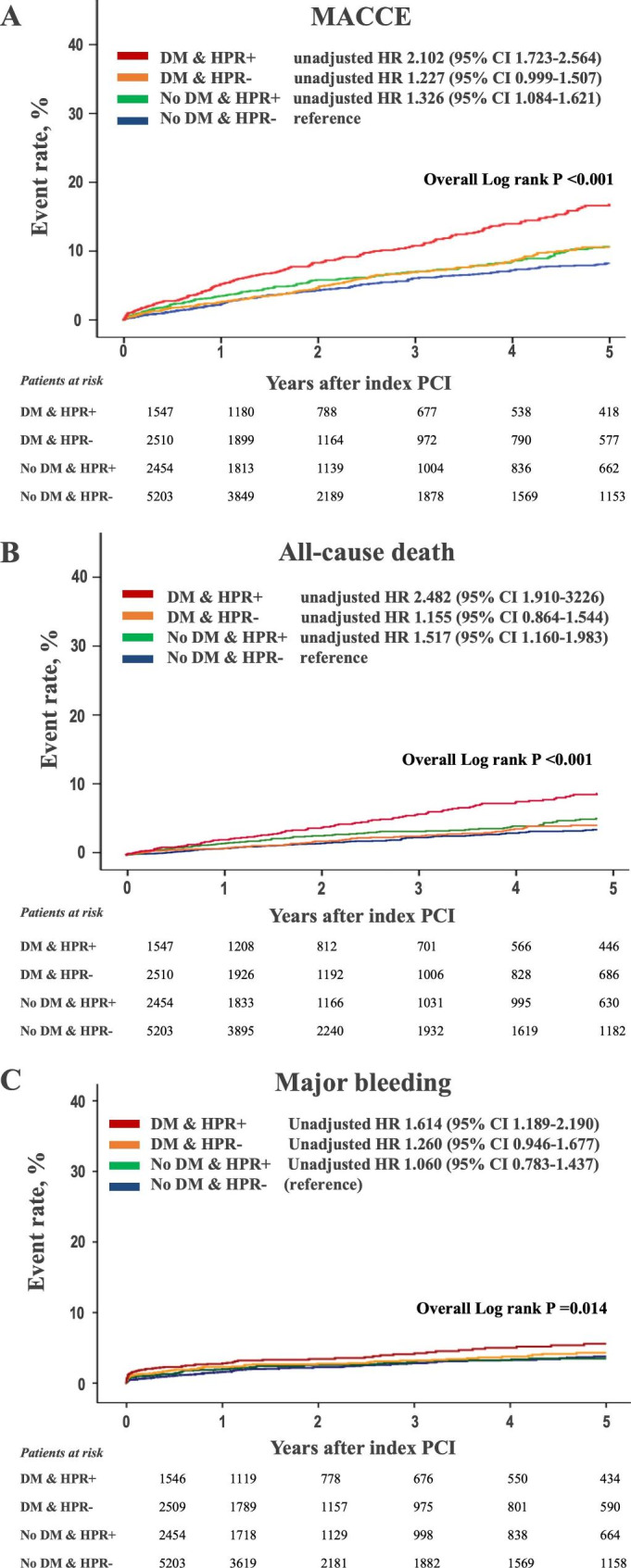
Kaplan–Meier curves according to presence of DM and HPR phenotype. **A** MACCE; **B** all-cause death; **C** major bleeding DM: diabetes mellitus; HPR: high platelet reactivity; HR: hazard ratio; MACCE: major cardiac and cerebrovascular event

We categorized patients into the four groups according to presence of DM and HPR phenotype. Both DM and HPR phenotype showed the similar prognostic implication in terms with MACCE occurrence. Figure 4 shows that the highest rates of MACCE and all-cause death were found in DM patients with HPR compared with other groups (vs. non-DM without HPR: unadjusted hazard ratio [HR]: 2.102; 95% CI: 1.723–2.564; *P <* 0.001). In multivariable analysis, only DM phenotype with HPR significantly increased the risk of MACCE compared to non-DM phenotype without HPR (HR: 1.607; 95% CI: 1.301–1.984; *P* < 0.001) (Table 3).

**Table 3:** Risk factors for MACCE occurrence

Variables	Multivariate analysis		
	Exp(β)	95% CI	*P*-value
**Age ≥ 75-year-old**	1.848	1.569–2.176	< 0.001
**Female**	1.340	1.140–1.575	< 0.001
**Body mass index ≥ 25 kg/m** ^**2**^	0.689	0.584–0.811	< 0.001
**Index presentation with AMI**	1.400	1.202–1.631	< 0.001
**Non-DM without HPR**	reference		
** Non-DM with HPR**	1.104	0.899–1.356	0.345
** DM without HPR**	1.097	0.890–1.352	0.386
** DM with HPR**	1.607	1.301–1.984	< 0.001
**Hypertension**	1.300	1.100–1.537	0.002
**Chronic kidney disease**	1.642	1.390–1.939	< 0.001
**Congestive heart failure**	1.404	1.103–1.788	0.006
**Anemia**	1.396	1.182–1.649	< 0.001
**Prior CVA**	1.623	1.301–2.025	< 0.001

AMI: acute myocardial infarction; CI: confidence interval; CVA: cerebrovascular accident; DM: diabetes mellitus; HPR: high platelet reactivity; MACCE: major adverse cardiac and cerebrovascular event

We evaluated prognostic impact of HPR according to DM status. In non-DM patients, HPR phenotype did not significantly increase the risk of MACCE (adjusted HR [HR_adj_]: 1.073; 95% CI: 0.869–1.325; *P* = 0.511), whereas HPR was an independent determinant for MACCE occurrence among diabetic subjects (HR_adj_: 1.507; 95% CI: 1.193–1.902; *P* < 0.001) (Supplement Table 1). In terms with all-cause death, HPR phenotype was significantly associated with the increased risk only in DM patients (HR_adj_: 1.805; 95% CI: 1.316–2.476; *P* < 0.001), but not in non-DM subjects (HR_adj_: 1.082; 95% CI: 0.818–1.430; *P* = 0.582). HPR phenotype significantly increased the risk of stent thrombosis irrespective of DM status (DM: HR_adj_, 2.956; 95% CI, 1.280–6.825; *P* = 0.011 and non-DM: HR_adj_, 3.259; 95% CI, 1.630–6.515; *P* < 0.001) (*P*_*interaction*_ = 0.869). However, there were no differences in the risk of major bleeding across the groups.

## Discussion

This study investigated the most extensive clinical data to evaluate the long-term prognostic impact of platelet reactivity according to DM status in CAD patients undergoing DES implantation. The major findings were as below: (1) platelet reactivity during clopidogrel treatment was higher in patients with diabetes than those without diabetes, which was related with insulin treatment and the severity of DM (Hb_A1c_ level); (2) HPR enhanced the risks of MACCE and all-cause death, which association appeared significant only in diabetic patients; (3) the risk of major bleeding was not associated with HPR phenotype; and (4) prognostic implication of diabetic status and HPR phenotype appeared similar, and its association showed the synergistic effect on MACCE rate.

DM itself is a well-established risk factor for CV events in patients undergoing PCI. The major pathophysiology of CV events is associated with its atherogenicity, and increased thrombogenicity in diabetic patients is associated with worse clinical outcomes. In addition, DM status has been associated with the level of platelet reactivity and the prevalence of HPR. Their platelets have dysregulated signaling pathways, that lead to a hyperreactive phenotype with enhanced adhesion, aggregation, and activity [26]. Hyperglycemia can increase platelet reactivity by inducing non-enzymatic glycation of proteins on the surface of the platelets. Such glycation decreases membrane fluidity and increases the propensity for platelets activation [14]. Insulin antagonizes the effect of platelet agonists such as collagen, ADP, epinephrine, and platelet-activating factor, which can induce high platelet reactivity [15]. Superoxide may increase platelet reactivity by enhancing intraplatelet release of calcium after activation and limiting the biological activity of nitric oxide (NO) [27, 28] Endothelial dysfunction also increases platelet reactivity by decreased production and the effect of NO and prostacyclin [17]. Because of these pathologic changes, responsiveness to P2Y_12_ inhibitor was decreased [29, 30] Our data showed that the level of platelet reactivity (i.e., PRU) was correlated with HbA1c level and the proportion of HPR increased according to Hb_A1c_ level. In other words, not only presence of diabetes, but also diabetic condition (e.g., Hb_A1c_ level or insulin treatment) affected platelet reactivity during P2Y_12_ inhibitor therapy. Therefore, strict control of diabetic condition (i.e., Hb_A1c_ < 6.5%) may affect the effect of antiplatelet regimens and decrease the rate of HPR, which may be related with a better clinical outcome in DES-treated patients.

HPR on antiplatelet therapy is a well-validated risk factor of ischemic events in patients undergoing PCI [13, 31–33]. The optimal cut-off values for HPR are different between the East Asian and Western populations, and it is known already that PRU values are higher in East Asian populations (218 PRU in PTRG-DES vs. 188 PRU in ADAPT-DES during clopidogrel treatment) [20, 31]. Although the distribution of PRU was shifted to the right side, the ischemic events rate after PCI was known to be lower in East Asians than Westerners. This was known as “East Asian Paradox” [34]. The present study already validated this concept by presenting ≥ 252 PRU as an optimal cut-off of HPR using time-dependent ROC curve analysis. It is quite higher than 208 PRU, which was suggested in Western population [12, 31]. The PRU values of diabetic patients were higher than those of non-diabetic patients, and PRU values gradually increased according to the severity of DM. In terms of clinical outcomes, this study showed that HPR and DM had a combined prognostic implication following DES implantation. Based on the change of the platelet reactivity in patients with diabetes, the clinical studies of potent P2Y_12_ inhibitor such as prasugrel [35] and ticagrelor [36] showed a favorable outcome following PCI in diabetic patients. It is interesting to note that in non-DM patients, although the incidence of MACCE is numerically higher in the HPR group, there is no statistically significant difference. This might be because the non-DM group, having fewer co-morbidities, presented fewer events, thus not showing a statistical distinction. Considering that diabetes itself is a strong risk factor for CV events after PCI, the risk of HPR for MACCE occurrence is also quite high even in East Asian patients. Choice of potent P2Y_12_ inhibitor can be more preferred in DM patients with poorly controlled glucose level or on insulin treatment.

This study had several limitations. First, the PTRG-DES consortium excluded patients treated with potent P2Y_12_ inhibitors. An added advantage could be that all patients were treated with the same drug (clopidogrel). As a result, it can guarantee the homogeneity of the study population. However, the resulting disadvantage is that the difference in the effect of potent P2Y_12_ inhibitor therapy for diabetic patients with HPR cannot be evaluated. Second, to assess the status of DM, detailed data on the degree of control, duration, type of medications, and the presence of diabetic complications are essential. Due to the limitations of a large cohort study, we cannot obtain detailed DM-related data for individual patients. However, we believe that Hb_A1c_ levels and insulin treatment are appropriate indicators of DM control and long-standing DM at the time of PFT. Finally, platelet function test and Hb_A1c_ level were assessed with each other at a single time-point measurement. Platelet reactivity can change according to the phase and may be linked with the status of glycemic control.

## Conclusions

This analysis from the PTRG-DES consortium including a large-scale East Asian patients demonstrated that glucose control affected the level of platelet reactivity during clopidogrel treatment. HPR phenotype and DM status showed the similar prognostic implication after DES implantation, and HPR was significantly associated with ischemic risk only in diabetic patients.

### Electronic supplementary material

Below is the link to the electronic supplementary material.

Supplementary Material 1

## Data Availability

PTRG-PFT data sets are not publicly available because of data protection agreements but can be available from the corresponding author on reasonable request.

## Abbreviations

AA: Arachidonic acid
AMI: Acute myocardial infarction
ARU: Aspirin reaction units
ASCVD: Atherosclerotic cardiovascular disease
BARC: Bleeding Academic Research Consortium
CAD: Coronary artery disease
CKD: Chronic kidney disease
CI: Confidence intervals
CV: Cardiovascular
DES: Drug-eluting stent
DM: Diabetes mellitus
Hb: Hemoglobin
HPR: High platelet reactivity
HR: Hazard ratio
IQR: Interquartile range
MACCE: Major adverse cardiac and cerebrovascular events (MACCE)
MACE: Major cardiovascular event
MI: Myocardial infarction
NO: Nitric oxide
OR: Odds ratio
PCI: Percutaneous coronary intervention
PFT: Platelet function test
PRU: P2Y12 reaction unit
SD: Standard deviation
ST: Stent thrombosis
