# Supplementary material for: Implication of diabetic status on platelet reactivity and clinical outcomes after drug-eluting stent implantation: results from the PTRG-DES consortium
Source: Cardiovasc Diabetol. 2023 Sep 7;22:245. doi: 10.1186/s12933-023-01976-4 (PMC10486029; doi:10.1186/s12933-023-01976-4)
Supplement: Supplementary file 1 — Supplementary Material 1 [file 12933_2023_1976_MOESM1_ESM.docx]

**Supplementary Fig. 1.** Study flow chart


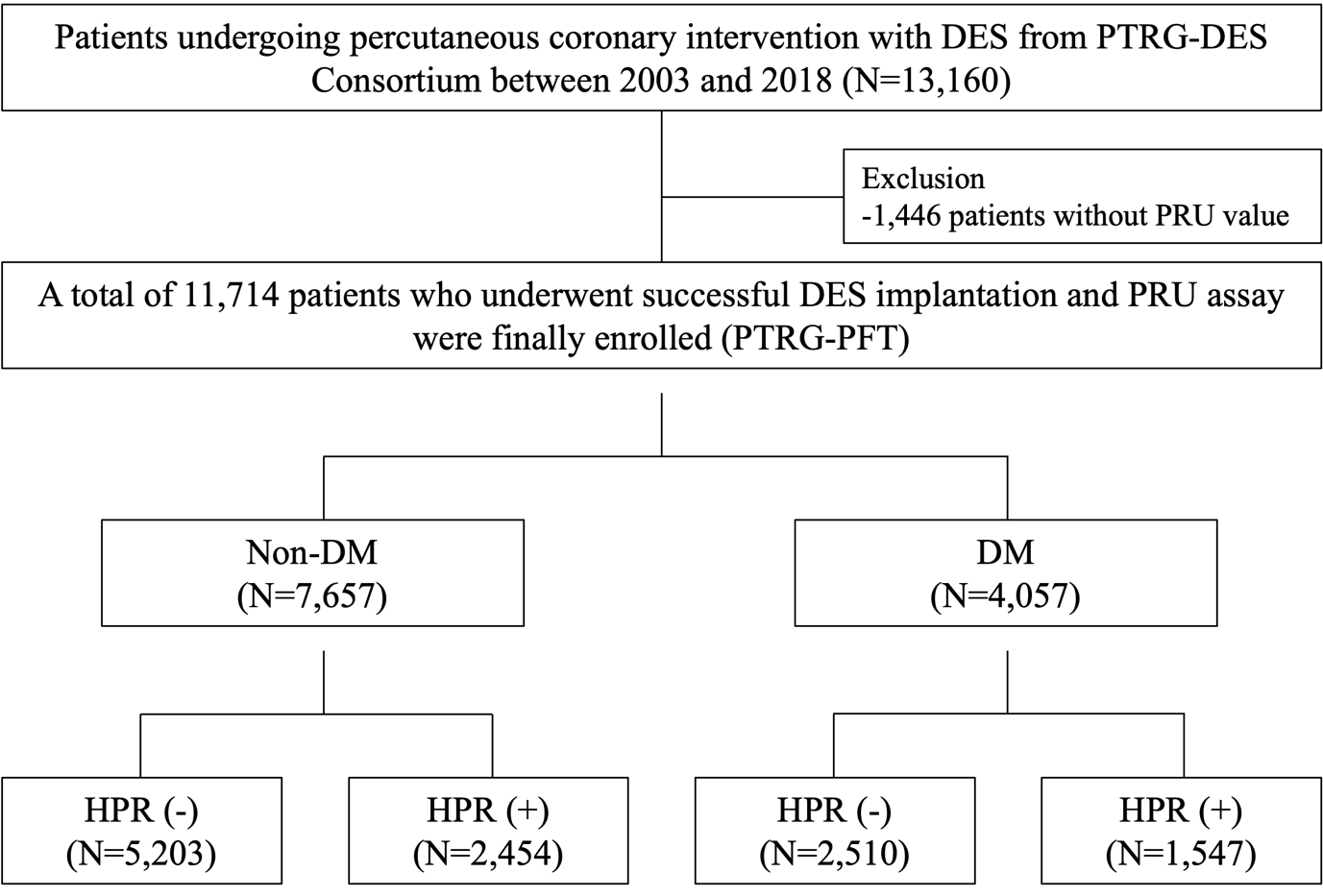


**Supplementary Fig. 2.** Platelet reactivity and HPR risk according to Hb_A1c_


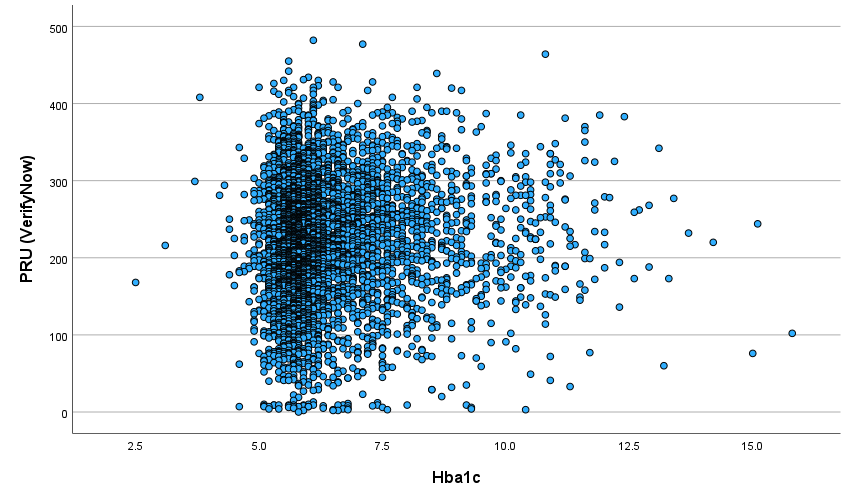


***r* = 0.065, *P*-value <0.001**


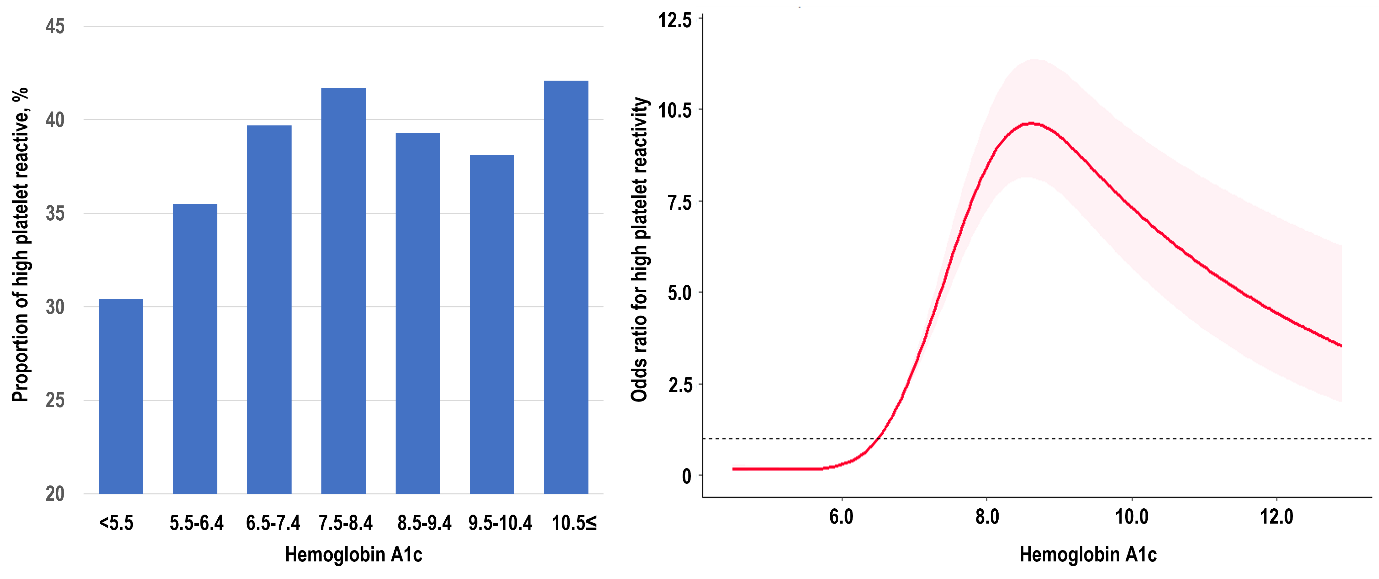


**Supplement Table 1.** Clinical outcomes according to DM presence and HPR

| **Event, n (%)** | **Non-DM** | | | | **DM** | | | | **Interaction**  ***P* – value** |
| --- | --- | --- | --- | --- | --- | --- | --- | --- | --- |
|  | **HPR (-)**  **(n = 5,203)** | **HPR (+)**  **(n = 2,454)** | **Adjusted HR** | **95% CI** | **HPR (-)**  **(n = 2,510)** | **HPR (+)**  **(n = 1,547)** | **Adjusted HR** | **95% CI** |  |
| **MACCE** | 240 (4.6) | 158 (6.4) | 1.073 | 0.869-1.325 | 147 (5.9) | 164 (10.6) | 1.507 | 1.193-1.902 | 0.066 |
| **Death** | 124 (2.4) | 94 (3.8) | 1.082 | 0.818-1.430 | 72 (2.9) | 102 (6.6) | 1.805 | 1.316-2.476 | 0.053 |
| **Non-fatal MI** | 58 (1.1) | 40 (1.6) | 1.265 | 0.829-1.929 | 43 (1.7) | 31 (2.0) | 0.894 | 0.552-1.449 | 0.753 |
| **Stent thrombosis** | 15 (0.3) | 21 (0.9) | 3.259 | 1.630-6.515 | 10 (0.4) | 16 (1.0) | 2.956 | 1.280-6.825 | 0.869 |
| **Non-fatal stroke** | 68 (1.3) | 31 (1.3) | 0.848 | 0.544-1.322 | 41 (1.6) | 41 (2.7) | 1.683 | 1.070-2.648 | 0.076 |
| **Major bleeding** | 123 (2.4) | 63 (2.6) | 0.773 | 0.564-1.061 | 76 (3.0) | 62 (4.0) | 0.980 | 0.708-1.425 | 0.346 |

DM = diabetes mellitus, HPR = high platelet reactivity, MACCE = major adverse cardiac and cerebrovascular events, MI = myocardial infarction.

**Supplement Table 2.** Baseline characteristics of study population according to DM and HRP

| **Variables** | **Overall**  **(n = 11,714)** | **Non-DM** | | **DM** | | ***P*-value** |
| --- | --- | --- | --- | --- | --- | --- |
|  |  | **HPR (-)**  **(n = 5,203; 44.4%)** | **HPR (+)**  **(n = 2,454; 20.9%)** | **HPR (-)**  **(n = 2,510; 21.4%)** | **HPR (+)**  **(n = 1,547; 13.2%)** |  |
| Index presentation, n (%) |  |  |  |  |  | <0.001 |
| Stable angina | 4910 (41.9) | 2092 (40.2) | 979 (39.9) | 1150 (45.8) | 689 (44.5) |  |
| Unstable angina | 3466 (29.6) | 1534 (29.5) | 723 (29.5) | 766 (30.5) | 443 (28.6) |  |
| Non-ST-segment elevation MI | 1860 (15.9) | 856 (16.5) | 396 (16.1) | 362 (14.4) | 246 (15.9) |  |
| ST-segment elevation MI | 1478 (12.6) | 721 (13.9) | 356 (14.5) | 232 ( 9.2) | 169 (10.9) |  |
| Age, years | 64.4 ± 10.9 | 62.4 ± 11.4 | 66.7 ± 10.7 | 64.3 ± 9.9 | 67.5 ± 9.7 | < 0.001 |
| Male, n (%) | 7951 (67.9) | 3896 (74.9) | 1410 (57.5) | 1812 (72.2) | 833 (53.8) | < 0.001 |
| Body mass index, kg/m^2^ | 24.5 ± 3.1 | 24.5 ± 3.0 | 24.2 ± 3.2 | 24.8 ± 3.1 | 24.7 ± 3.2 | < 0.001 |
| Risk factors, n (%)* |  |  |  |  |  |  |
| Hypertension | 7049 (60.2) | 2722 (52.3) | 1450 (59.1) | 1740 (69.3) | 1137 (73.5) | < 0.001 |
| Dyslipidemia | 7555 (64.5) | 3385 (65.1) | 1495 (60.9) | 1637 (65.2) | 1038 (67.1) | < 0.001 |
| Smoking | 3285 (28.0) | 1669 (32.1) | 572 (23.3) | 755 (30.1) | 289 (18.7) | < 0.001 |
| Diabetes mellitus | 4057 (34.6) | 0 (0.0) | 0 (0.0) | 2510 (100.0) | 1547 (100.0) | < 0.001 |
| Insulin-treated | 367 (3.1) | 0 (0.0) | 0 (0.0) | 153 (6.1) | 120 (7.8) | < 0.001 |
| Chronic kidney disease | 2432 (20.8) | 781 (15.0) | 511 (20.8) | 581 (23.1) | 559 (36.1) | < 0.001 |
| Current dialysis | 162 (1.4) | 25 (0.5) | 36 (1.5) | 41 (1.6) | 60 (3.9) | < 0.001 |
| Anemia | 2921 (24.9) | 792 (15.2) | 826 (33.7) | 618 (24.6) | 685 (44.3) | < 0.001 |
| Previous history, n (%) |  |  |  |  |  |  |
| History of peripheral artery disease | 1453 (12.4) | 567 (10.9) | 319 (13.0) | 310 (12.4) | 257 (16.6) | <0.001 |
| History of congestive heart failure | 880 (7.5) | 394 (7.6) | 161 ( 6.6) | 223 ( 8.9) | 102 ( 6.6) | 0.008 |
| Previous MI | 839 (7.2) | 341 (6.6) | 163 ( 6.6) | 216 ( 8.6) | 119 ( 7.7) | 0.006 |
| Previous PCI | 1568 (13.4) | 620 (11.9) | 286 (11.7) | 407 (16.2) | 255 (16.5) | < 0.001 |
| Previous CABG | 150 (1.3) | 54 (1.0) | 17 (0.7) | 63 (2.5) | 16 (1.0) | < 0.001 |
| Previous stroke | 813 (6.9) | 304 (5.8) | 163 (6.6) | 212 (8.4) | 134 (8.7) | < 0.001 |
| Laboratory measurements |  |  |  |  |  |  |
| VerifyNow PRU | 217.8 ± 78.7 | 172.4 ± 56.4 | 300.8 ± 39.3 | 179.0 ± 55.2 | 301.4 ± 39.3 | < 0.001 |
| LV ejection fraction, | 58.8 ± 10.6 | 59.2 ± 10.1 | 58.9 ± 10.2 | 58.2 ± 11.2 | 57.9 ± 11.5 | < 0.001 |
| WBC, x10^3^/mm^3^ | 7.9 ± 3.0 | 7.9 ± 3.1 | 7.6 ± 3.0 | 7.9 ± 2.9 | 7.8 ± 2.8 | 0.303 |
| Hemoglobin, g/dL | 13.6 ± 1.8 | 14.1 ± 1.7 | 13.0 ± 1.6 | 13.7 ± 1.9 | 12.5 ± 1.8 | < 0.001 |
| Platelet, x10^3^/mm^3^ | 233.6 ± 72.4 | 233.7 ± 70.0 | 234.3 ± 72.1 | 233.8 ± 75.9 | 232.3 ± 75.3 | 0.644 |
| GFR, mL/min/1.73 m^2^ (MDRD) | 78.7 ± 27.1 | 82.0 ± 24.6 | 78.4 ± 26.4 | 77.5 ± 28.8 | 69.5 ± 30.6 | < 0.001 |
| HbA_1c_, | 6.6 ± 1.4 | 6.0 ± 0.8 | 6.1 ± 0.9 | 7.5 ± 1.6 | 7.5 ± 1.5 | < 0.001 |
| Total cholesterol, mg/dL | 174.0 ± 44.5 | 179.6 ± 43.8 | 177.4 ± 44.2 | 166.4 ± 43.8 | 165.2 ± 42.9 | < 0.001 |
| LDL-cholesterol, mg/dL | 106.8 ± 43.4 | 110.3 ± 37.8 | 109.6 ± 38.7 | 99.5 ± 36.1 | 98.0 ± 36.3 | < 0.001 |
| HDL-cholesterol, mg/dL | 44.0 ± 12.8 | 44.6 ± 12.0 | 44.7 ± 12.1 | 42.6 ± 10.9 | 42.1 ± 11.6 | < 0.001 |
| Triglyceride, mg/dL | 143.2 ± 98.3 | 145.2 ± 105.0 | 131.6 ± 86.7 | 149.3 ± 97.9 | 145.2 ± 91.2 | 0.355 |
| Angiographic feature |  |  |  |  |  |  |
| ACC/AHA lesion, n (%) |  |  |  |  |  | < 0.001 |
| A/B1 type | 5238 (44.7) | 2438 (46.9) | 1071 (43.6) | 1088 (43.3) | 641 (41.4) |  |
| B2/C type | 6476 (55.3) | 2765 (53.1) | 1383 (56.4) | 1422 (56.7) | 906 (58.6) |  |
| Number of diseased vessels, n (%) |  |  |  |  |  | < 0.001 |
| One | 7170 (61.2) | 3356 (64.5) | 1484 (60.5) | 1452 (57.8) | 878 (56.8) |  |
| Two | 3039 (25.9) | 1290 (24.8) | 636 (25.9) | 701 (27.9) | 412 (26.6) |  |
| Three | 1505 (12.8) | 557 (10.7) | 334 (13.6) | 357 (14.2) | 257 (16.6) |  |
| Multivessel disease, n (%) | 4544 (38.8) | 267 (5.1) | 124 (5.1) | 111 (4.4) | 70 (4.5) | < 0.001 |
| Bifurcation lesion, n (%) | 1363 (11.6) | 542 (10.4) | 302 (12.3) | 315 (12.5) | 204 (13.2) | 0.003 |
| Chronic total occlusion lesion, n (%) | 821 (7.0) | 370 (7.1) | 126 (5.1) | 204 (8.1) | 121 (7.8) | < 0.001 |
| Procedural data |  |  |  |  |  |  |
| Multivessel PCI, n (%) | 2917 (24.9) | 1194 (22.9) | 586 (23.9) | 694 (27.6) | 443 (28.6) | < 0.001 |
| Treated lesions, n (%) |  |  |  |  |  |  |
| Left main coronary artery | 572 (4.9) | 267 (5.1) | 124 (5.1) | 111 (4.4) | 70 (4.5) | 0.490 |
| Left anterior descending artery | 6960 (59.4) | 3112 (59.8) | 1420 (57.9) | 1494 (59.5) | 934 (60.4) | 0.336 |
| Left circumflex artery | 3434 (29.3) | 1465 (28.2) | 705 (28.7) | 766 (30.5) | 498 (32.2) | 0.008 |
| Right coronary artery | 4460 (38.1) | 1867 (35.9) | 962 (39.2) | 1015 (40.4) | 616 (39.8) | < 0.001 |
| PCI for left main or  left anterior descending artery, n (%) | 7327 (62.5) | 3292 (63.3) | 1498 (61.0) | 1565 (62.4) | 972 (62.8) | 0.304 |
| Stent type, n (%) **^†^** |  |  |  |  |  | < 0.001 |
| 1^st^ generation DES | 944 (8.1) | 368 ( 7.1) | 247 (10.1) | 182 ( 7.3) | 147 ( 9.5) |  |
| 2^nd^ generation DES | 10770 (91.9) | 4835 (92.9) | 2207 (89.9) | 2328 (92.7) | 1400 (90.5) |  |
| Number of stent, n | 1.6 ± 0.8 | 1.6 ± 0.8 | 1.6 ± 0.8 | 1.6 ± 0.8 | 1.7 ± 0.8 | < 0.001 |
| Stent length, mm | 35.9 ± 22.5 | 35.0 ± 22.1 | 34.9 ± 22.2 | 37.5 ± 23.0 | 37.8 ± 23.6 | < 0.001 |
| Stent diameter, mm | 3.02 ± 0.44 | 3.0 ± 0.5 | 3.0 ± 0.4 | 3.0 ± 0.4 | 3.0 ± 0.4 | < 0.001 |
| Concomitant medications, n (%) |  |  |  |  |  |  |
| Aspirin | 11409 (97.4) | 5078 (97.6) | 2400 (97.8) | 2433 (96.9) | 1498 (96.8) | 0.091 |
| Clopidogrel | 11714 (100.0) | 5,203 (100) | 2,454 (100) | 2,510 (100) | 1,547 (100) | ns |
| Cilostazol | 1219 (10.4) | 499 ( 9.6) | 263 (10.7) | 297 (11.8) | 160 (10.3) | 0.024 |
| Beta blocker | 6669 (56.9) | 3009 (57.8) | 1427 (58.1) | 1363 (54.3) | 870 (56.2) | 0.014 |
| Angiotensin blockade | 6927 (59.1) | 2956 (56.8) | 1437 (58.6) | 1527 (60.8) | 1007 (65.1) | < 0.001 |
| Calcium channel blocker | 2817 (24.0) | 1193 (22.9) | 568 (23.1) | 643 (25.6) | 413 (26.7) | 0.003 |
| Statin | 10379 (88.6) | 4667 (89.7) | 2165 (88.2) | 2189 (87.2) | 1358 (87.8) | 0.006 |
| Proton pump inhibitor | 1991 (17.0) | 878 (16.9) | 469 (19.1) | 361 (14.4) | 283 (18.3) | < 0.001 |

Continuous variables were expressed in mean ± SD or median (IQR) as indicated.

ACC = American College of Cardiology; AHA = American Heart Association; CABG = coronary artery bypass graft; DES = drug eluting stent; GFR = glomerular filtration rate; HbA_1c_ = hemoglobin A_1c_; HDL = high density lipoprotein; LDL = low density lipoprotein; LV = left ventricular; MDRD = Modification of Diet in Renal Disease; MI = myocardial infarction; PCI = percutaneous coronary intervention; PRU = P2Y12 Reaction Unit; WBC = white blood cell.

*Hypertension was diagnosed by one of the followings: (1) history of hypertension diagnosed and treated with medication, diet and/or exercise; (2) blood pressure greater than 140 mmHg systolic or 90 mmHg diastolic on at least 2 occasions; or (3) currently on antihypertensive pharmacologic therapy.; Dyslipidemia was diagnosed by one of followings: (1) total cholesterol ≥ 200 mg/dl; (2) LDL cholesterol ≥ 130 mg/dl; (3) HDL cholesterol < 40 mg/dl; or (4) triglycerides ≥ 150 mg/dl; Current smoker was defined as use of tobacco within one year of this admission; Diabetes mellitus was diagnosed by one of the followings: (1) a history of diabetes, regardless of duration of disease, or need for antidiabetic agents; (2) a fasting blood glucose ≥ 126 mg/dl; or (3) glycosylated hemoglobin ≥ 6.5; Chronic kidney disease was diagnosed by one of the followings: (1) GFR < 60 mL/min/1.73m^2^ (MDRD); (2) on dialysis; or (3) history of a renal transplantation; Anemia was defined as hemoglobin level < 12 g/dl in women and 13 g/dl in men.

**^†^**First-generation DES indicated durable polymer-based paclitaxel-eluting stents (PES: Taxus, Pico) or sirolimus-eluting stent (SES: Cypher); Second-generation DES indicated next-generation DESs including everolimus-eluting stent (EES: Promus, Xience), zotarolimus-eluting stent (ZES: Endeavor, Resolute, Onyx), biolimus-eluting stent (BES: Biolimus A9), and plymer-free SES.; If a patient were treated with first- and second-generation DESs together, this patient was considered as implantation with first generation DES.
